# Atypical Analysis of a Graphite-Based Anode Prepared Using Aqueous Processes

**DOI:** 10.3390/molecules30193947

**Published:** 2025-10-01

**Authors:** Kuan-Yi Liao, Chia-Chin Chang, Yuh-Lang Lee, Ten-Chin Wen

**Affiliations:** 1 Department of Chemical Engineering, National Cheng Kung University, Tainan 70101, Taiwan; morley23133463@gmail.com; 2 Department of Greenergy, National University of Tainan, Tainan 70005, Taiwan; ccchang@mail.nutn.edu.tw; 3 Graduate Institute of Energy and Sustainability Technology, National Taiwan University of Science and Technology, Taipei City 10607, Taiwan

**Keywords:** atypical analysis, graphite anode, aqueous process, break-in

## Abstract

In order to form a solid electrolyte interphase (SEI) layer using aqueous processes, a graphite anode called MG-AQP was designed by wrapping and crosslinking graphite particles with aqueous composites (AQCs), which contained zwitterionic polymer, zwitterion molecules, and lithium salts. First, MG-AQP was used to fabricate a full lithium-ion battery (LIB) cell with Li[Ni_0.8_Mn_0.1_Co_0.1_]O_2_ (NMC811) as the cathode, denoted as LIB-MG-AQP//NMC811, to demonstrate its performance via a 0.5 C-rate break-in and 1 C-rate cycling. Accordingly, this showed that LIB-MG-AQP exhibits outstanding cyclic stability. To evaluate its electrochemical performance, MG-AQP and lithium metal were used to fabricate a half cell named LIBs-MG-AQP. According to the initial cyclic voltammetry curve, almost no surface reaction for forming an SEI layer exists in LIBs-MG-AQP, illustrating its high initial coulombic efficiency of 92% at a 0.5 C-rate break-in. These outstanding results are due to the fact that the AQC has fewer cracks, thus blocking solvent molecules from passing from the electrolyte into the graphite anode. This study provides new insights to optimize graphite anodes via 0.5 C-rate break-in rather than conventional SEI formation to save time and energy.

## 1. Introduction

Conventionally, graphite is used as an anode material for lithium-ion batteries (LIBs) due to its availability, stability, and good conductivity [1,2,3,4,5,6]. Carboxymethyl cellulose (CMC), commonly employed as a binder for graphite anodes, provides high viscosity and strong adhesion, enabling uniform slurry dispersion and improved particle cohesion [7,8,9]. However, there are still some drawbacks with the usage of CMC as a binder for graphite anodes [10].

For instance, under formation processes, many side products are formed, such as LiF, Li_2_CO_3_, LiOH, ROCO_2_Li, and ROLi in solid electrolyte interface (SEI) layers, as well as the generation of C_2_H_4_, CO_2_, H_2_, and HF gases due to irreversible reactions between lithium ions, solvents, and anionic molecules with electron transfer [11,12,13,14]. Consequently, a significant number of lithium ions are consumed during the initial process. Moreover, the formation of weak SEI layers occurs in graphite anodes with the application of conventional electrolytes, as reported in some studies [15,16,17,18]. Alternatively, graphite anodes prepared with a CMC binder exhibit pronounced surface cracks, which can be attributed to the tendency of linear CMC polymers to aggregate into clusters during the drying process, leading to portions of the graphite surface becoming exposed and cracked. In this case, under high C-rates, lithium ions favor the low resistance pathways provided by the cracks instead of traveling through the intact electrode surfaces. Nevertheless, the weakness of the SEI layer becomes so severe that many lithium ions are consumed in the reforming of the SEI layer rather than contributing to reversible energy storage, resulting in poor performance and cyclic stability. Generally, in the conventional design of graphite anodes, it is necessary to form an SEI layer at 0.1 C-rate. Also demonstrated in previous studies is the fact that due to the crystallization properties of CMC at low temperatures, lithium ions with extremely high resistances travel from the electrolyte to the graphite surface to undergo energy storage. The above statements show the many drawbacks of CMC being used for graphite anodes.

To address the limitations of conventional binders in graphite anodes, in our previous studies, CMC was reacted with sulfobetaine methacrylate (SBMA) as a zwitterionic monomer, and zwitterion molecules were added to obtain a product denoted as CMC-SBMA^a^, which was applied as a graphite binder [19,20]. It has been observed that zwitterion molecules can improve the formation of a strong SEI layer comprising rich LiF. Moreover, zwitterion molecules can enhance the transportation of lithium ions with high diffusivities and low resistances. The strong interactions between the zwitterion molecules can reduce the crystallization tendency of CMC-SBMA^a^, enabling this binder to deliver excellent performance at low temperatures. Thus, the addition of zwitterion molecules provides superior performance and cyclic stability in graphite anodes.

However, it is inevitable that some cracks occur on graphite anodes produced using CMC-SBMA^a^ as a binder due to the linear polymer and zwitterion molecules. At a high C-rate, solvent molecules carrying lithium ions can penetrate these cracks and reach the graphite surface, where they participate in surface reactions that produce a weak and highly resistive SEI [21,22]. In this study, we solve this problem by developing a novel aqueous fabrication method for the development of graphite anodes. First, aqueous composites containing the zwitterionic polymer CMC-SBMA^a^, zwitterion molecules, and lithium salts with crosslinkers, which form the network structure to wrap the graphite particles, are used to develop anodes for lithium-ion batteries (LIBs). We hypothesize that the aqueous composites will prevent cracks from forming on the graphite surface and block solvent molecules from passing from the electrolyte to the graphite anode. Accordingly, this study illustrates how our aqueous processes save time in the SEI formation of graphite anodes. The electrochemical performance of the anode at 0.5 C as a high C-rate break-in is atypically analyzed and the results are described below.

## 2. Results and Discussion

### 2.1. Demonstration of Full Cells

**Figure 1:**
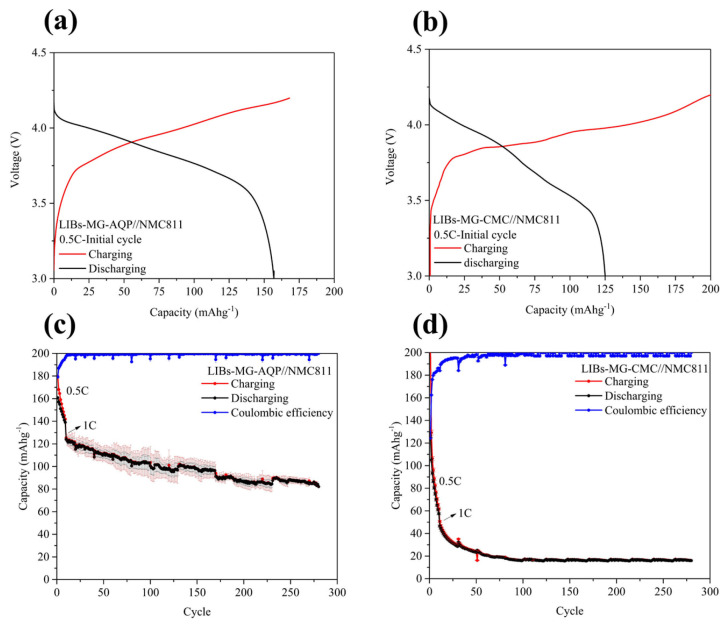
The first charging and discharging curves of (**a**) LIBs-MG-AQP//NMC811 and (**b**) LIBs-MG-CMC//NMC811 at 0.5 C-rate break-in. The cyclic stabilities of (**c**) LIBs-MG-AQP//NMC811 and (**d**) LIBs-MG-CMC//NMC811 for 280 cycles.

In this study, MG-AQP and MG-CMC as the anode and Li[Ni_0.8_Mn_0.1_Co_0.1_]O_2_(NMC811) as the cathode were used to fabricate LIBs in full-coin cells for LIBs-MG-AQP//NMC811 and LIBs-MG-CMC//NMC811, respectively. Both LIBs-MG-AQP//NMC811 and LIBs-MG-CMC//NMC811 undergo a break-in process at a 0.5 C-rate under atypical analysis; their charging and discharging curves are shown in Figure 1a,b. The curve of NMC811 under a 0.5 C-rate break-in is presented in Figure S1. Accordingly, the initial curve of LIBs-MG-AQP//NMC811 shows a smoother slope than that of LIBs-MG-CMC//NMC811. LIBs-MG-AQP//NMC811 and LIBs-MG-CMC//NMC811 exhibit voltage plateaus at 3.84 V and 3.81 V at SOC 50% during the discharging process, representing the lower resistances of LIBs-MG-AQP//NMC811 than LIBs-MG-CMC//NMC811. The charging and discharging capacities of LIBs-MG-AQP//NMC811 are 168.7 mAhg^−1^ and 157 mAhg^−1^, while those of LIBs-MG-CMC//NMC811 are 200 mAhg^−1^ and 125.04 mAhg^−1^. The initial coulombic efficiencies of LIBs-MG-AQP//NMC811 and LIBs-MG-CMC//NMC811 are 92% and 63%, respectively. For the cyclic stabilities after 10 cycles at a 1 C-rate, as shown in Figure 1c,d, the coulombic efficiencies of LIBs-MG-AQP//NMC811 become stable at nearly 100%. Conversely, MG-CMC//NMC811 exhibits stable capacities and coulombic efficiencies after 70 cycles. For the final cycles, LIBs-MG-AQP//NMC811 and LIBs-MG-CMC//NMC811 remain at 83% and 25% at 280 cycles. It is observed that the numerous surface reactions required for forming an SEI take place in MG-CMC//NMC811 during the break-in process at a 0.5 C-rate, substantially reducing the amount of lithium ions for energy storage. Therefore, MG-CMC//NMC811 is necessary to form the SEI layer under a low C-rate, as shown in Figure S2. However, LIBs-MG-AQP//NMC811 exhibits excellent stability during the break-in process at a 0.5 C-rate, indicating its high and stable capacity during cycling analysis. Therefore, it seems that the new insights obtained from the development of aqueous graphite anodes can be used to demonstrate the energy and time saved by these devices. A detailed electrochemical analysis of graphite anodes is presented below.

### 2.2. Electrochemical Performance of Half Cells

**Figure 2:**
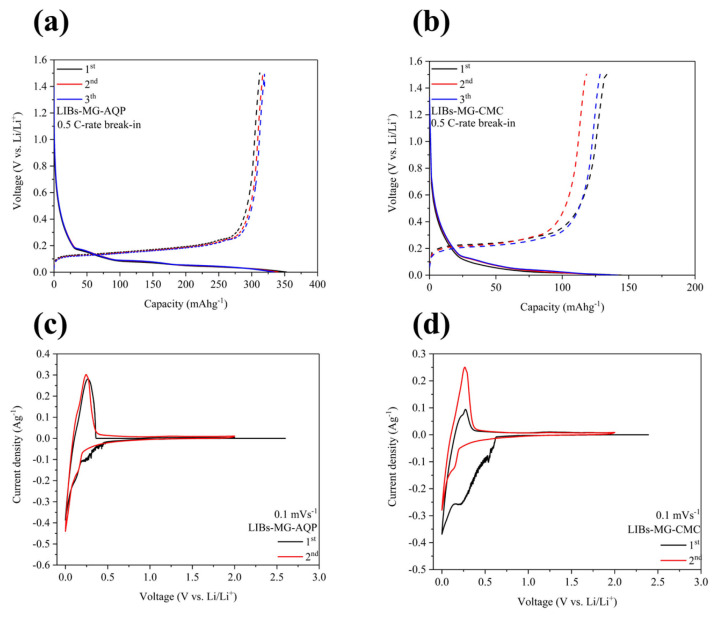
The first lithiation and delithiation curves of (**a**) LIBs-MG-AQP and (**b**) LIBs-MG-CMC at a 0.5 C-rate break-in. The initial cyclic voltammetry curves of (**c**) LIBs-MG-AQP and (**d**) LIBs-MG-CMC.

To present the electrochemical performance of the half cells fabricated in an anode containing the lithium metals LIBs-MG-AQP and LIBs-MG-CMC at a 0.5 C-rate break-in, charging and discharging curves are shown in Figure 2a,b. The initial coulombic efficiencies of LIBs-MG-AQP and LIBs-MG-CMC are 88.44% and 73.7%, which are nearly in line with the efficiency values for full cells of LIBs-MG-AQP//NMC811 and LIBs-MG-CMC//NMC811. Additionally, the voltage values at SOC 50% are 0.172 and 0.275 V vs. Li/Li^+^ during the initial delithiation process, demonstrating that lower resistances exist in LIBs-MG-AQP than in LIBs-MG-CMC. These results also correspond to the performance obtained from the CV analysis shown in Figure 2c,d. As can be observed from Figure 2c,d, initially, many surface reactions occur in LIBs-MG-CMC from 0.63 V vs. Li/Li^+^ to 0.214 V vs. Li/Li^+^ during the lithiation process, while LIBs-MG-AQP exhibits only few surface reactions from 0.48 V vs. Li/Li^+^ to 0.215 V vs. Li/Li^+^, indicating that it is efficient for suppressing and stagnating the surface reactions when forming an SEI from solvent molecules with lithium ions. During the delithiation process, the anodic peak for LIBs-MG-AQP is positioned more toward the left than that for LIBs-MG-CMC, indicating the lower energy consumption of LIBs-MG-AQP than LIBs-MG-CMC. The following figures show the discharging and charging curves at 1C-rate cycling as well as Nyquist plots of both LIBs-MG-AQP and LIBs-MG-CMC to demonstrate the behavior of resistance.

**Figure 3:**
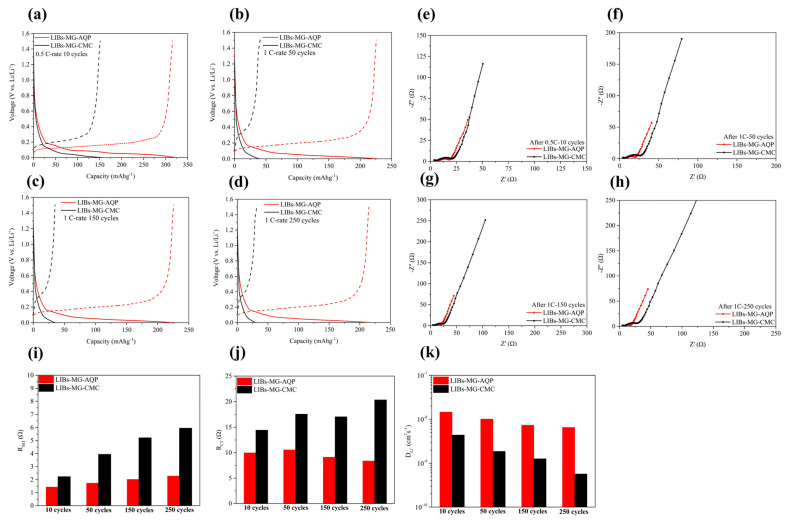
Curves showing the voltage versus capacity of LIBs-MG-AQP and LIBs-MG-CMC at (**a**) a 0.5 C-rate of 10 cycles, (**b**) a 1 C-rate of 50 cycles, (**c**) a 1 C-rate of 150 cycles, and (**d**) a 1 C-rate of 250 cycles. Nyquist plots of LIBs-MG-AQP and LIBs-MG-CMC at (**e**) a 0.5 C-rate of 10 cycles, (**f**) a 1 C-rate of 50 cycles, (**g**) a 1 C-rate of 150 cycles, and (**h**) a 1 C-rate of 250 cycles. The values of (**i**) R_SEI_, (**j**) R_SEI_, and (**k**) D_Li_^+^ for LIBs-MG-AQP and LIBs-MG-CMC at a 0.5 C-rate of 10 cycles, a 1 C-rate of 50 cycles, a 1 C-rate of 150 cycles, and a 1 C-rate of 250 cycles.

The discharging and charging curves of both LIBs-MG-AQP and LIBs-MG-CMC before cycling and at 1 C-rate cycling at 50, 150, and 250 cycles are presented in Figure 3a–d. LIBs-MG-CMC exhibits increasing voltage plateaus under 1 C-rate cycling during the delithiation process from 0.36 V vs. Li/Li^+^ at 50 cycles to 0.43 vs. Li/Li^+^ at 250 cycles. By contrast, there are almost no changes in the voltage plateau for LIBs-MG-AQP from 0.208 V vs. Li/Li^+^ at 50 cycles to 0.213 V vs. Li/Li^+^ at 250 cycles. Correspondingly, the Nyquist plots of LIBs-MG-AQP and LIBs-MG-CMC before cycling and under 1 C-rate cycling at 50, 150, and 250 cycles are presented in Figure 3e–h. To quantify the values of resistances, the data for the resistance of the SEI (R_SEI_), interfacial charge transfer resistance (R_CT_), and lithium ion diffusion coefficient (D_Li_^+^), calculated using Equation (1), during cycling are presented in Figure 3i–k.

(1) $$D_{{Li}^{+}}={\left(\frac{RT}{{\sqrt{2}F}^{2}AC_{{Li}^{+}}\times W}\right)}^{2}$$

where $D_{{Li}^{+}}$ (cm^2^$s^{-1}$) denotes the diffusivities of lithium in the anode; n is the charge transfer number; F (C ${mol}^{-1}$) is Faraday’s constant of 96,485; A (cm^2^) is the area of the circle-shaped anode; $C_{{Li}^{+}}$ (mol cm^−3^) is the concentration of lithium ion in bulk; R is the gas constant for 8.314 × 10^3^ (kJ ${\left(mol\;K\right)}^{-1}$); *T* is the temperature (K); and W ($\Omega s^{-0.5})$ is the Walberg coefficient.

It is observed that the R_SEI_ and R_CT_ values increase in LIBs-MG-CMC after 250 cycles. However, for LIBs-MG-AQP, these values remain almost the same from 50 cycles to 250 cycles. Moreover, the D_Li_^+^ values for LIBs-MG-AQP maintain almost the same values, while those for LIBs-MG-CMC continue dropping up to 250 cycles. In this case, many zwitterion molecules and lithium salts contained within the AQC undergo mutual dissociation to form the free lithium ions and unassembled zwitterion molecules that provide an adequate pathway to improve mobility and diffusivity. The AQC is also designed for application in graphite anodes, preventing solvent molecules from passing from the electrolyte to the graphite anodes, which can improve the coulombic efficiency and stability.

### 2.3. SEM Images of Graphite Anodes

**Figure 4:**
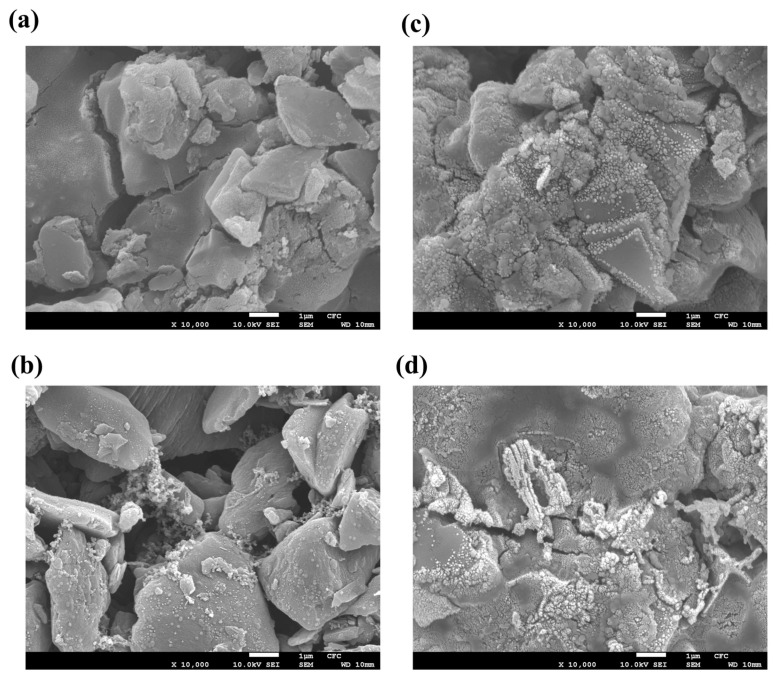
SEM images of (**a**) MG-AQP and (**b**) MG-CMC anodes before cycling, and (**c**) MG-AQP and (**d**) MG-CMC after 280 cycles.

SEM images of MG-AQP and MG-CMC before and after cycling are presented in Figure 4a–d, respectively. Figure 4a,b show that the morphologies of graphite particles in MG-AQP is smoother than in MG-CMC before cycling. Additionally, the more uniform dispersions for graphite and binder present in MG-AQP and MG-CMC are also visible. Figure 4c,d, representing MG-AQP and MG-CMC after cycling, show that MG-CMC exhibits a considerably greater number of large cracks than MG-AQP. It is obvious that the many cracks on the graphite in MG-CMC contribute to the persistently substantial consumption of lithium ions, which form the SEI layer, which corresponds to the low coulombic efficiency of LIBs-MG-CMC//NMC811. Conversely, due to the network structure and lack of cracks in the AQC, the lithium ions in MG-AQC are mostly used for energy storage rather than the formation of the SEI. Nevertheless, MG-AQP still exhibits a few cracks before cycling, as the sharp edges of the graphite cannot be completely wrapped by the AQC. In this case, at a high current rate, there is still a risk that lithium ions accompanied by solvent molecules preferentially penetrate through these cracks into the inner graphite, resulting in extensive surface reactions with the solvent and reduced cycling stability. Accordingly, this has driven us to utilize more spheroidal graphite in anodes and ensure that it is wrapped more uniformly in future research in order to achieve more superior performance.

### 2.4. XPS Analysis of Graphite Anodes

**Figure 5:**
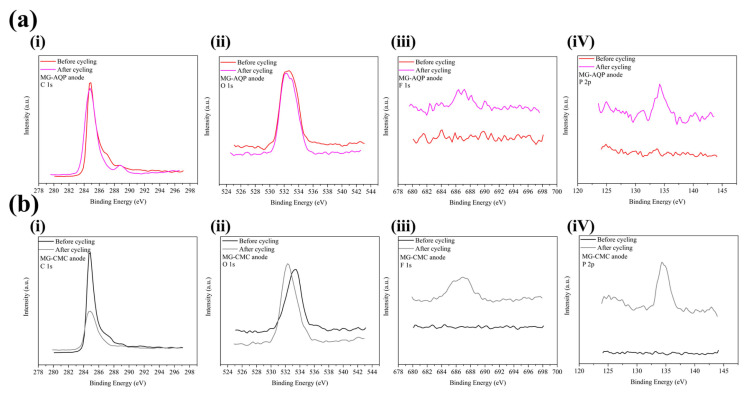
The XPS analysis for (**a**) MG-AQP and (**b**) MG-CMC of (**i**) C, (**ii**) O, (**iii**) F, and (**iV**) F elements before and after cycling.

The XPS curves of the C, O, F, and P elements from the MG-AQP and MG-CMC anodes before and after cycling are presented in Figure 5a,b in subfigures (i), (ii), (iii), and (iV), respectively. In Figure 5a, the curves of the C and O element of MG-AQP nearly overlap before and after cycling, suggesting that the graphite particles are not covered by a thick SEI layer and no significant O-rich SEI species have formed. This indicates that solvent molecules cannot be transported through our designed AQC. As shown in Figure 5a (iii) and (iV), some F- and P-based species have formed around the graphite particles, showing that a few PF_6_^−^ species participate in the strong formation of LiF and Li_3_PO_4_ at the SEI. By contrast, according to Figure 5b, the C and O spectra exhibit suppressed peak intensities and significant peak shifts, indicating the formation of thick SEI layers. In addition, F- and P-based species are more prominently detected on the particle surfaces. These XPS results clearly demonstrate that the AQC composites effectively suppress excessive solvent decomposition and facilitate the formation of thin, stable, and inorganic-rich SEI layers (LiF and Li_3_PO_4_). In contrast, the conventional CMC binder induces the accumulation of thick, unstable SEI films. The differences between the XPS results for the MG-AQP and MG-CMC anodes are consistent with the above electrochemical performance, indicating that the MG-AQP anodes exhibit high initial coulombic efficiency and undergo minimal surface reactions with solvent molecules for forming the SEI. The findings also suggest that almost no cracks are present on the MG-AQP surfaces, as further corroborated by the following analyses.

### 2.5. Property Differences Between AQC and CMC

**Figure 6:**
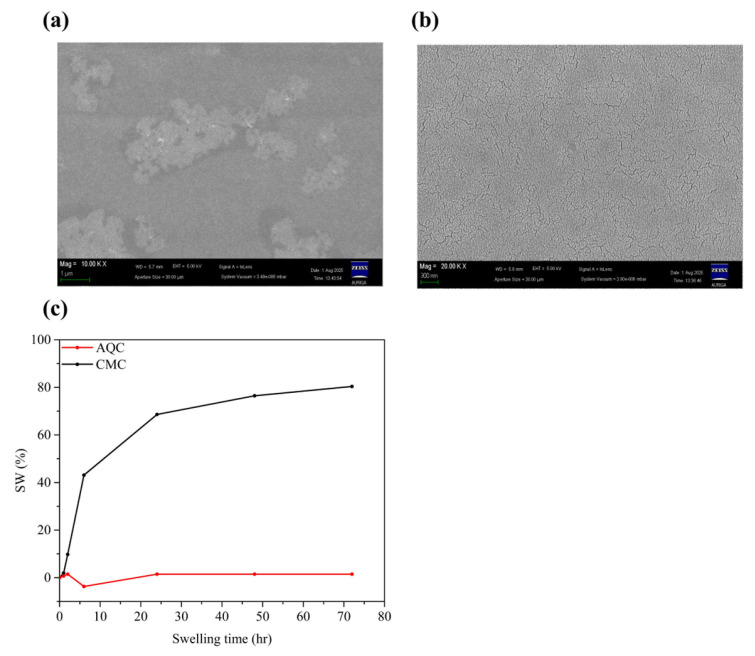
SEM images of (**a**) AQC and (**b**) CMC, as well as (**c**) SW ratio versus time for AQC and CMC.

Finally, the properties of both the AQC and CMC were found to coincide with the results from the SEM images of MG-AQP and MG-CMC. First, the SEM images of the AQC and CMC are presented in Figure 6a,b. It can be observed that AQC possesses few cracks on the surface, while there are significant large cracks on the CMC, indicating that many solvent molecules could penetrate through the cracks. The swelling ratio (SW) versus time for both the AQC and CMC with DMC solvent are presented in Figure 6c. After three days, the SWs of the AQC and CMC are 1.47% and 80.39%, indicating that the solvent molecules are not more permeable through the AQP than the CMC. These results provide convincing evidence that applying the AQC to graphite anodes at a break-in of 0.5 C-rate can replace that of 0.1 C-rate SEI formation.

**Figure 7:**
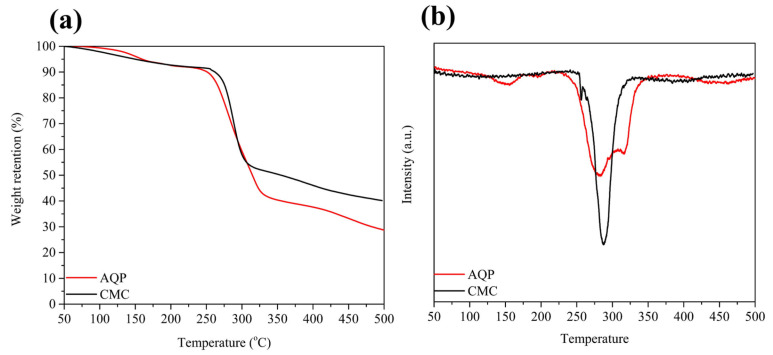
(**a**) The TGA curves and (**b**) dTGA curves of the AQC and CMC from 50 °C to 500 °C.

In terms of the thermal stabilities of the AQC and CMC, Figure 7a,b show that the temperatures of the dehydration process used for the decomposition of AQC and CMC range from 250 °C to 350 °C and from 255 °C to 320 °C. It seems that the AQC presents lower temperatures for thermal decomposition than CMC. However, as can be seen from the differential TGA (dTGA) curves in Figure 7b, the AQC exhibits a broader curve and higher temperature on the final day of the dehydration process than CMC, indicating a slower rate of heat release. It is possible that the use of AQCs in graphite prevents the thermal runaway of LIBs.

**Figure 8:**
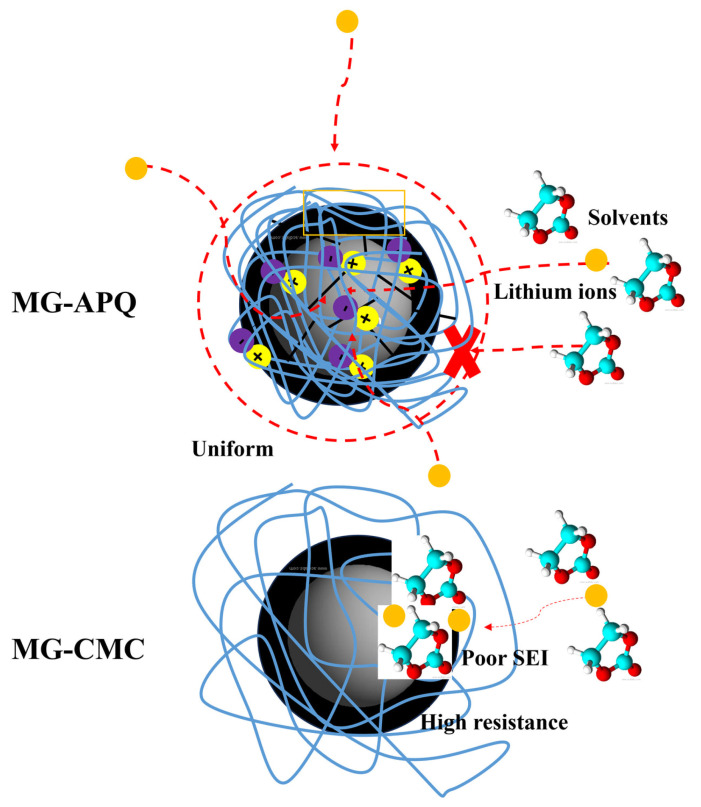
Schematic diagram of the MG-AQP and MG-CMC mechanisms as an atypical analysis.

The mechanism of the AQCs and CMC used to fabricate the graphite anode is presented in Figure 8. The large numbers of dissociated zwitterion molecules and lithium ions in the AQC provide many uniform pathways for ion transportation, improving diffusivity and lowering resistance. Compared to conventional CMC binders, graphite anodes with AQCs can exhibit lower energy consumption during charging or lithiation but higher energy during discharging or delithiation. Additionally, in our previous studies, zwitterion molecules were found to improve the dispersion of slurries, thus saving more power when fabricating graphite anodes compared to CMC-based graphite anodes. Moreover, due to the fewer cracks in the AQC, solvent molecules do not travel from the electrolyte to the graphite anode. Therefore, it is not necessary to form an SEI layer conventionally at a 0.1 C-rate. Instead, the 0.5 C-rate break-in used in this study is treated as an atypical analysis to show that LIBs-MG-AQP//NMC811 exhibits excellent initial coulombic efficiency and cyclic stability for saving energy as well as time. Furthermore, this work highlights the need for in situ spectroscopy such as Raman spectra, XRD analysis, and molecular dynamics simulations, which will be presented in future studies.

## 3. Materials and Methods

### 3.1. Materials

Carboxymethyl cellulose (CMC, BVH8) was purchased from Ashland Inc. (authorized distributor of Ashland Inc., Taipei, Taiwan). Sulfobetaine methacrylamide (SBMA), lithium trifluoromethanesulfonate (LiCF_3_SO_3_) (LiOTf), and glutaraldehyde (GA, 25 wt%) were purchased from Sigma-Aldrich (via Merck Ltd. Taiwan, Taipei, Taiwan). Lithium hydroxide hydrate (LiOH·H_2_O, 98 wt%) and betaine (99 wt%) were purchased from Thermo Scientific (via DKSH Taiwan, Taipei, Taiwan). Dimethyl carbonate (DMC, 99 wt%) was purchased from Alfa Aesar (via DKSH Taiwan, Taipei, Taiwan). Graphite (MG30) was provided from China Steel Chemical Corporation, Kaohsiung, Taiwan. Super P was provided by Timcal (via Taiwan Maxwave Co., Ltd., Taipei, Taiwan). Li[Ni_0.8_Mn_0.1_Co_0.1_]O_2_ (NMC811) (∼95.5%) was purchased from Ningbo Ronbay New Energy, Ningbo, China. Polyvinylidene fluoride (PVdF-5130) powder was purchased from Solvay S.A. (via a subsidiary of Solvay S.A, Taipei, Taiwan). Methylpyrrolidone (NMP) was purchased from the uniregion bio-tech company, New Taipei City, Taiwan.

### 3.2. Preparation of Aqueous Composites

In accordance with our previous study, first, LiOH and SBMA were added to 2 wt% of CMC aqueous solution equimolarly. CMC was reacted with SBMA at 50 °C for 16 h through oxa-Michael addition to obtain the CMC-SBMA^a^ product. Subsequently, aqueous composites (AQCs) were prepared via mixing CMC-SBMA^a^ with betaine and LiOTf equimolarly based on the amount of SBMA in the AQC.

### 3.3. Preparation of Graphite Anode

MG30, as one type of graphite, was wrapped using aqueous composites. MG30 particles were mixed in the aqueous composites evenly at a mass ratio of 128.24:1 (MG30 particles/AQC), and then wrapping the particles tightly, the aqueous composites were crosslinked into a network structure, being formed at 50 °C by adding glutaraldehyde (GA, 1 wt% relative to CMC-SBMA^a^) as a crosslinker. After the crosslinking reaction, 1wt% Super P, 0.75 wt% aqueous composites and 1.32 wt% styrene–butadiene rubber (SBR) were added to the solution with 55 wt% of solid content to prepare the anode slurry. Subsequently, a copper clad laminate was coated with graphite slurry with a weight film thickness of 200 μm. Finally, this graphite anode, called MG-AQP, was obtained for use in fabricating LIBs. The copper-clad laminate with graphite slurry was dried at 100 °C, vacuum-dried overnight, and pressed using a rolling machine.

For comparison, the conventionally fabricated graphite slurry comprised 96.18 wt% MG30 with 1.5 wt% CMC, 1wt% Super P, and 1.32 wt% styrene–butadiene rubber (SBR), with 55 wt% solid content in 45 wt% of water as a solvent. Similar to the above preparation, the graphite anode, denoted as MG-CMC, was obtained.

### 3.4. Preparation of NMC811 Cathode Slurry

The NMC811 cathode comprised 95.5 wt% of NMC811 particles, 2.5 wt% of PVdF, and 2 wt% of Super P. Similar to the graphite anode slurries, 55 wt% solid content in 45 wt% of NMP was used as a solvent. The obtained cathode was used to fabricate LIBs after the aluminum-clad laminate covered with NMC811 slurry was dried at 100 °C, vacuum-dried overnight, and pressed using a rolling machine [23,24].

### 3.5. Fabrication of the LIBs

MG-AQP and MG-CMC, in circle shapes with a diameter of 13 mm, were used to fabricate LIBs with lithium metal in the coin cells, called LIBs-MG-AQP and LIBs-MG-CMC, respectively. Here, 1M LiPF_6_ in EC/DMC (1:1) as a liquid electrolyte, infiltrating fiber-reinforced plastic as a separator, was sandwiched between the graphite anodes and lithium metal. Information regarding LIBs-MG-AQP and LIBs-MG-CMC is provided in Table S1.

Furthermore, in order to demonstrate the actual performance of the LIBs, both MG-AQP and MG-CMC as anodes with Li[Ni_0.8_Mn_0.1_Co_0.1_]O_2_(NMC811) as a cathode were used to fabricate the LIBs for LIBs-MG-AQP//NMC811 and LIBs-MG-CMC//NMC811, whose information is provided in Table S2. Similarly, 1M LiPF_6_ in EC/DMC (1:1) as an electrolyte, infiltrating fiber-reinforced plastic as a separator, was sandwiched between the graphite anodes and NMC811 cathodes.

### 3.6. Characterization

#### 3.6.1. Analysis of Aqueous Composites and CMC

Both AQC and CMC were analyzed by a thermo-gravimetric analyzer (TGA, Perkin Elmer TGA4000; Waltham, MA, USA) from 50 to 500 °C with 2 °C min^−1^ to obtain their thermal properties. Images of the aqueous composites and CMC were also obtained by a scanning electron microscope (SEM) with Pt sputter coating to observe the morphologies at 10 kV.

Swelling analyses of the AQC and CMC were performed to determine whether solvent molecules diffuse into the materials. In this study, the aqueous composite and CMC were dipped into a DMC solvent. The weights of the aqueous composite and CMC were measured at various times to determine the swelling ratios, as per Equation (2).

(2) $$SW\;(t)=\frac{w\;(t)-w_{0}}{w_{0}}\times 100$$

where SW (*t*) represents the swelling ratio, w represents the weight of the membranes, and $w_{0}$ represents the weight of the dry membranes.

#### 3.6.2. Analysis of Half-Coin Cells

Both LIBs-MG-AQP and LIBs-MG-CMC were broken in with a 0.5 C-rate from 1.5 V to 0 V using a battery charge–discharge tester (BAT-750B) for ten cycles as an atypical analysis. In addition, LIBs-MG-AQP and LIBs-MG-CMC were analyzed by cyclic voltammetry (CV) with a scan rate of 0.1 mVs^−1^ from 2 V to 0 V to observe their electrochemical behavior under the break-in process. Then, they were analyzed at a 1 C-rate for cyclic stability. Here, we chose 10, 50, 150, and 250 cycles for LIBs-MG-AQP and LIBs-MG-CMC, which were analyzed by electrochemical impedance spectroscopy (EIS) with a lithium-ion state of charge of 0 (SOC = 0). After cycling, the graphite anodes from the LIBs-MG-AQP and LIBs-MG-CMC were analyzed by SEM to observe their morphologies. X-ray photoelectron spectroscopy (XPS) was employed to analyze the graphite anodes of the LIBs-MG-AQP and LIBs-MG-CMC before and after cycling, in order to clarify the formation of the SEI.

#### 3.6.3. Analysis of Full-Coin Cells

Similarly, the 0.5 C-rate as the break-in process for 10 cycles was applied for both LIBs-MG-AQP//NMC811 and LIBs-MG-CMC//NMC811. After 10 cycles, the stability of the LIBs-MG-AQP//NMC811 and LIBs-MG-CMC//NMC811 was analyzed at a 1 C-rate for 250 cycles.

## 4. Conclusions

In this study, we designed aqueous composites, called AQCs, which contain zwitterionic polymers, zwitterion molecules, and lithium salts. Graphite, wrapped using an AQC with a crosslinker, was used to fabricate the anode via aqueous processes, denoted as MG-AQP. The atypical analysis of its electrochemical performance demonstrates that a 0.5 C-rate break-in achieves an excellent initial coulombic efficiency of 92% and long-term cycle stability of 83% retention after 280 cycles for LIBs-MG-AQP//NMC811. For half cells, LIBs-MG-AQP shows fewer surface reactions according to CV analysis, as well as low and unchangeable resistance after cycling. Structural and physicochemical analyses coincided with the results of electrochemical performance, indicating that the AQC exhibits minimal cracking, thus limiting solvent penetration. These observations indicate that the AQC both physically blocks solvent access to graphite, reducing SEI-forming reactions, and chemically facilitates ion transportation via the dissociation of lithium ions and zwitterion molecules. This study highlights a novel approach for saving time and energy when producing excellent graphite anodes. Moreover, this strategy offers a practical pathway toward the time- and energy-efficient activation of LIBs, suggesting its potential for sustainable and scalable graphite anode fabrication for LIBs in the future.

## Data Availability

The data presented in this study are available on request.

## Supplementary Materials

The following supporting information can be downloaded at: https://www.mdpi.com/article/10.3390/molecules30193947/s1. Table S1: Information on coin cells for LIBs-MG-AQP and LIBs-MG-CMC; Table S2: Information on coin cells for LIBs-MG-AQP//NMC811 and LIBs-MG-CMC//NMC811; Figure S1: The initial delithiation and lithiation curves of NMC811 at a 0.5 C-rate; Figure S2: (a) The initial charging and discharging curves and (b) cyclic stability of LIBs-MG-CMC//NMC811 at a 1 C-rate.
