# Supplementary material for: Atypical Analysis of a Graphite-Based Anode Prepared Using Aqueous Processes
Source: Molecules. 2025 Oct 1;30(19):3947. doi: 10.3390/molecules30193947 (PMC12526116; doi:10.3390/molecules30193947)
Supplement: Supplementary file 1 [file molecules-30-03947-s001.zip › molecules-3874791-supplementary.pdf]

# Atypical analysis of graphite-based anode prepared by aqueous processes

## Supplementary Material

Kuan-Yi Liao [a], Chia-Chin Chang [b],

Yuh-Lang Lee [a*], Ten-Chin Wen [a*]

[a] Department of Chemical Engineering, National Cheng Kung University, Tainan 70101, Taiwan

[b] Department of Greenergy, National University of Tainan, Tainan, 70101, Taiwan

[c] Graduate Institute of Energy and Sustainability Technology, National Taiwan University of Science and Technology

[*]E-mail: yllee@mail.ncku.edu.tw, tcwen@mail.ncku.edu.tw

Table S1 Information of coin cells for LIBs-MG-AQC and LIBs-MG-CMC

| | LIBs-MG-AQP | LIBs-MG-CMC |
|---|---|---|
| Anode Area ($cm^2$) | 1.33 | 1.33 |
| Loading mass (mg) | 4.10 | 5.20 |
| Thickness (mm) | 0.02 | 0.025 |
| Density ($g/cm^3$) | 1.541 | 1.56 |
| Acticated mass (mg) | 3.94 | 5 |

Table S2 Information of coin cells for LIBs-MG-AQC//NMC811 and LIBs-MG-CMC//NMC811

| | LIBs-MG-AQP//NMC811 | | LIBs-MG-CMC//NMC811 | |
|---|---|---|---|---|
| | Anode | cathode | Anode | cathode |
| electrode Area ($cm^2$) | 1.33 | 1.33 | 1.33 | 1.33 |
| Loading mass (mg) | 7.4 | 14.9 | 11.7 | 19.1 |
| Thickness (mm) | 0.035 | 0.035 | 0.05 | 0.04 |
| Density ($g/cm^3$) | 1.59 | 3.20 | 1.759398496 | 3.59 |
| Acticated mass (mg) | 7.12 | 14.23 | 11.23 | 18.24 |
| N/P ratio | 1.16 | | 1.43 | |

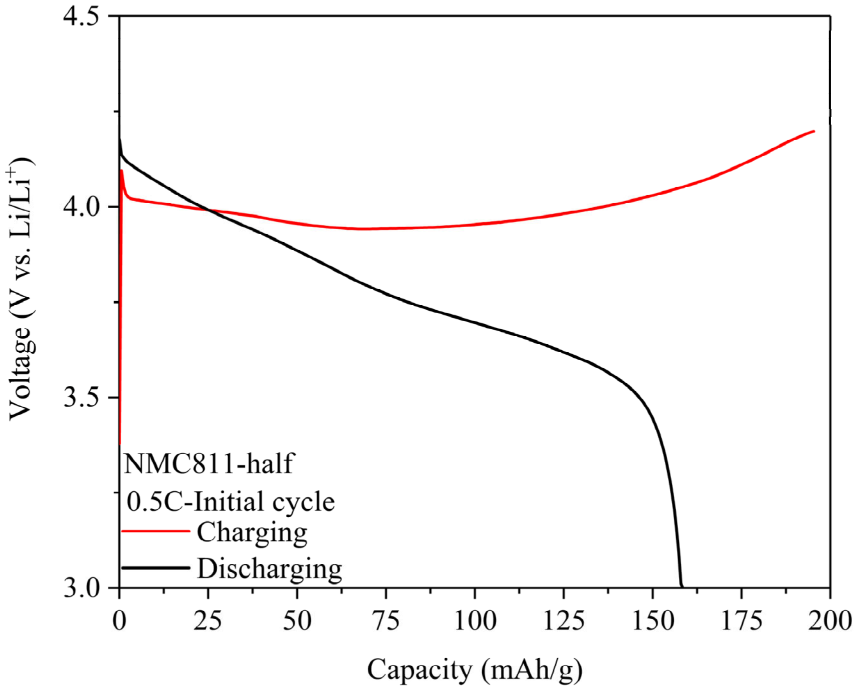


Figure S1 The initial delithiation and lithiation curves of NMC811 at 0.5 C-rate

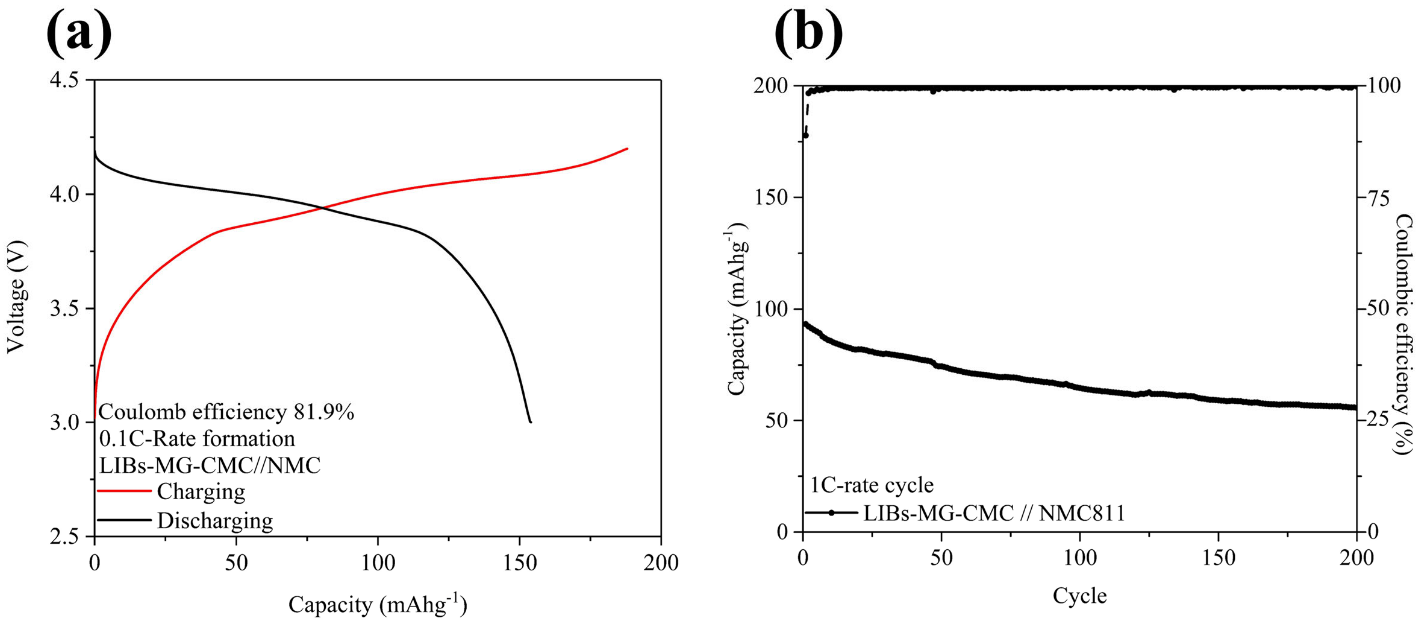


Figure S2 (a) The initial charging and discharging curves (b) cyclic stability of LIBs-MG-CMC//NMC811 at 1 C-rate
